# Feasibility and Usability of an mHealth App (mLab+) to Guide Users Through HIV and Syphilis Self-Testing: Pilot Randomized Controlled Trial

**DOI:** 10.2196/72955

**Published:** 2025-12-30

**Authors:** Maeve Brin, Thomas F Scherr, Janejira Chaiyasit, Jianfang Liu, Maura Abbott, Robert Garofalo, Lisa M Kuhns, Tess Sky, Ian Esliker, Rebecca Schnall

**Affiliations:** 1 School of Nursing, Columbia University, 560 W 168 Street, New York, NY, 10032, United States, 1 212-342-6886; 2 Vanderbilt University, Nashville, United States; 3 MGH Institute of Health Professions School of Nursing, Washington DC, United States; 4 Division of Adolescent & Young Adult Medicine, Ann & Robert H. Lurie Children’s Hospital of Chicago, Chicago, IL, United States; 5 Department of Pediatrics, Feinberg School of Medicine, Northwestern University, Chicago, IL, United States; 6 Mailman School of Public Health, Columbia University, New York, NY, United States

**Keywords:** HIV, syphilis, self-test, mobile health, mHealth, sexually transmitted infections, men who have sex with men, MSM

## Abstract

**Background:**

HIV self-testing is an important strategy in the US Ending the HIV Epidemic initiative. To facilitate uptake of self-testing, we developed the mLab app, which complements existing self-test options to support the potential for higher uptake of the HIV self-test. Syphilis, a sexually transmitted infection with currently rising prevalence and overlap in risk profiles with HIV, could similarly benefit from the advantages of companion diagnostic mobile apps such as mLab. Due to the success of the mLab app in promoting HIV self-testing during a randomized controlled trial and the scientific evidence of need for at-home syphilis testing, our team developed the mLab+ app, which supports both HIV and syphilis testing through an image processing algorithm that incorporates a duplex HIV and syphilis point-of-care test.

**Objective:**

We conducted a pilot study to assess the feasibility and usability of the mLab+ app for HIV and syphilis testing.

**Methods:**

We recruited participants who were assigned male sex at birth and reported sex with another man. Participants came to the Nurse Practitioner Group clinic for baseline and follow-up visits. Participants rated the usability of the app using the Health Information Technology Usability Evaluation Scale and the Post-Study System Usability Questionnaire at their 3-month follow-up visit. The primary outcome was the number of participants who were able to self-administer the DPP HIV-Syphilis test with the assistance of the mLab+ app. Feasibility was measured through recruitment pace, retention over 3 months, app usability, and paradata.

**Results:**

Of the 20 participants, 19 identified as male and 1 identified as nonbinary. Most participants (n=16) were able to complete the DPP HIV-Syphilis test with facilitation support from the mLab+ app. The average duration of an app session, from after authentication until log-out or abandonment, was 30 minutes and 33 seconds (SD 21 minutes and 40 seconds). Apart from the 27% (13/48) of sessions that were 5 minutes or less, the distribution of session durations was approximately normal. Users spent the longest time viewing testing screens (ie, timer screens, initial testing screen, test guided walkthroughs, test results, and picture and result upload). The overall mean scores on the Post-Study System Usability Questionnaire (2.65, SD 1.06) and Health Information Technology Usability Evaluation Scale (3.62, SD 1.07) indicated medium to high usability. The retention rate for the 3-month trial was 80% (16/20).

**Conclusions:**

The findings support the use of the mLab+ app as a tool for assisting consumers in self-testing for HIV and syphilis. The limitations of the study design warrant further examination outside of clinic settings to better understand the utility of these tools for improving consumer health outcomes.

## Introduction

HIV self-testing is recommended for diverse populations worldwide [1] and is considered an important strategy in the US Ending the HIV Epidemic initiative [2,3]. HIV self-tests can facilitate access to antiretroviral therapy, HIV preexposure prophylaxis (PrEP), and other prevention services [4,5]. Aligned with this evidence, and to facilitate uptake of HIV self-testing, our study team developed the mLab app through user-centered design including a youth advisory board and usability testing with informatics experts and end users [6]. The mLab app provides HIV prevention information, notification reminders for testing, step-by-step instructions for using OraQuick HIV tests, and an image upload function so individuals can send an image of their OraQuick HIV test for automated analysis. Individuals report their visual result of the OraQuick test on the app; the app then interprets the results of the OraQuick tests independent of the visually reported result. If an individual tests positive for HIV on the OraQuick HIV test, either via self-reported visual result or through mLab’s image processing functionality, the study team is notified and coordinates follow-up confirmatory testing with the participant.

The mLab app has advantages over stand-alone self-test options and supports the potential for higher uptake of the HIV self-test [7]. It addresses many of the current barriers to self-testing kits to overcome ambiguous test interpretations and provide immediate result reporting. Further, the mLab user interface promotes a holistic testing experience as it provides step-by-step error checking with clear picture instructions for completing the self-test. Although rapid tests such as the OraQuick seem simple to interpret, weak positive bands and weak control lines sometimes make it difficult for users to accurately interpret the test results, which we found in our own research on the OraQuick home test [8]. Another study found a correct interpretation rate of 70% for weak positives and 59% for invalid tests [9]. Beyond support for HIV testing, the mLab app provides information for those who test positive for HIV and educates users on the importance of follow-up testing and prevention services for those who test negative to promote linkage to care.

Implementation of HIV self-testing also provides an opportunity for self-testing of another sexually transmitted infection (STI) that has seen recent increases in prevalence in the United States: syphilis. From 2018 to 2022, there was an 80% increase in reported syphilis cases in the United States [10]. The rate of syphilis acquisition has increased steadily since 2000, especially among men, and men who have sex with men (MSM) constitute a disproportionate number of cases (36% of all cases in 2021 were among MSM) [11,12]. Moreover, the rate of syphilis among MSM is 100 times higher than that in men who have sex with women and even higher among young MSM [13,14]. In 2023, the highest rates of syphilis acquisition in male individuals were among men between the ages of 20 and 39 years (with rates per 100,000 ranging between 44.5% and 62% within each age subdivision) [15]. Additionally, disproportionate rates of HIV and STIs among transgender women and nonbinary people suggest the need for greater access to self-testing among these populations as well [16-20].

Importantly, syphilis makes it easier to both acquire and transmit HIV, and approximately half of MSM who have syphilis are coinfected with HIV [14,21]. The biological nature of syphilis, specifically syphilitic ulcer proliferation, facilitates transmission of HIV [22]. In addition, syphilis infections among people living with HIV are associated with higher HIV viral load and lower CD4 cell counts and, therefore, worsen the severity of HIV-related symptoms [23].

Prior studies have shown the need for at-home testing options for MSM and transgender women, not only for HIV but for STIs as well due to testing frequency recommendations and concerns about privacy and confidentiality [20,24,25]. Considering the higher level of coinfection with syphilis among people living with HIV, there is a need for increased concurrent testing for HIV and syphilis. One study showed that uptake of concurrent HIV and syphilis testing in a health care setting was highest among people between the ages of 25 and 34 years, suggesting potential acceptability of concurrent testing among this age group [26]. Moreover, the study acknowledged the social barriers to concurrent HIV and syphilis testing acceptance within the health care setting and called for the development of interventions that support decision-making between emergency department staff and high-risk patients [26].

**Figure 1.**
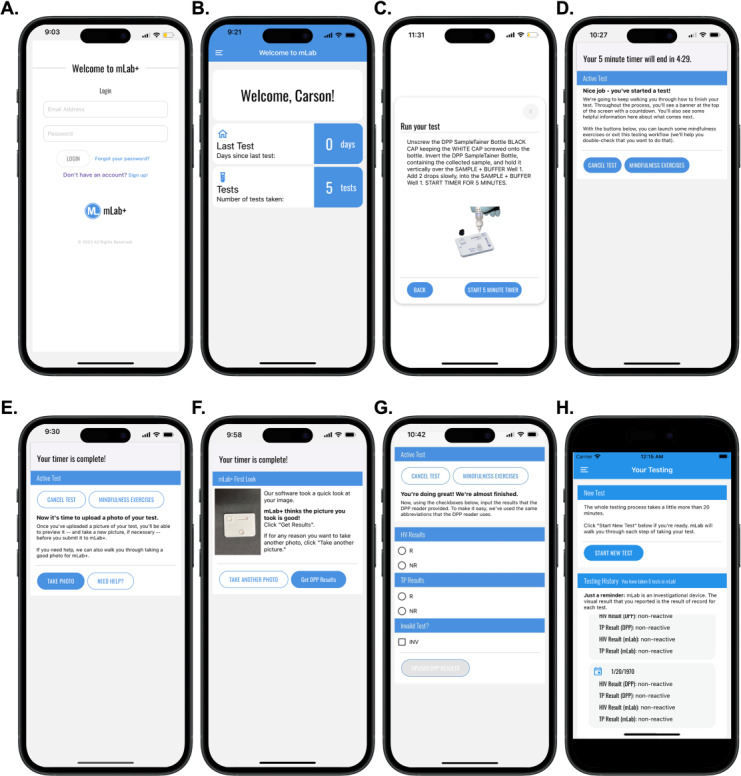
Screenshots from the mLab+ app: (A) authentication, (B) home or landing page, (C) step-by-step guided walkthrough of taking the test, (D) in-app timer with mindfulness exercises, (E) automatic timer-based navigation and upload of a photo of the test, (F) automated image processing review of photograph quality, (G) upload of user-reported visual result, and (H) test result history. This figure does not include information on any participants enrolled in the study.

Due to the success of the mLab app in a randomized controlled trial (RCT) and the scientific evidence of need for at-home syphilis point-of-care (POC) testing among young MSM and young transgender women, our team developed the mLab+ app (Figure 1), which supports HIV and syphilis testing at the POC. It facilitates the use of a duplex HIV and syphilis POC test and has an image processing algorithm that automatically and objectively interprets the diagnostic test. We conducted a rigorous pilot study to assess the feasibility and usability of the mLab+ app. The primary objective of this study was to evaluate the use of the mLab+ app in a clinical setting with 20 participants assigned male sex at birth who reported sex with a man and identified as male, transgender, or nonbinary. The aim of this study was to demonstrate the feasibility and usability of the mLab+ app to support users through HIV and syphilis self-testing.

## Methods

### Study Design

We conducted a 3-month pilot RCT with 40 participants from October 2023 to June 2024. Participants in both arms received STI counseling and condoms (baseline) and completed a survey (baseline and 3-month follow-up). Participants who were randomized to the intervention arm (mLab+ app) were given app access and took an HIV and syphilis test while using the app at both their baseline and 3-month follow-up appointments. This paper only reports the findings of the intervention arm.

### Recruitment Procedures

We used a multimodal recruitment strategy. We recruited through sexual networking apps (eg, Grindr and Scruff) and through community partners. Online advertisements included images of diverse MSM and were targeted to MSM through dating apps. A public link to a screener form was embedded within the advertisement image so that participants could fill out a brief survey to determine eligibility prior to being contacted by the research team. Regarding recruitment through community partners, research staff visited several clinics, organizations, gay bars, and shops throughout New York City to post physical flyers. Physical flyers included the study team’s phone number and a QR code that directed interested participants to the public screener link. The eligibility criteria can be found in Textbox 1. Eligible participants came to the Columbia University Irving Medical Center Nurse Practitioner Group (NPG) clinic for their study visit. Because all study procedures took place in person, participants were all from the New York City metropolitan area. They had to be able to travel to the study office for visits to be enrolled in the study.

Textbox 1. **Eligibility criteria for participation in the mLab+ app study.**

**Inclusion criteria**

- Between 18 and 39 years of age
- Assigned male sex at birth
- Ability to understand and read English
- Self-identifying as any race or ethnicity
- Substantial risk of acquiring HIV per Centers for Disease Control and Prevention guidance (eg, sexual partner with HIV, recent bacterial sexually transmitted disease, high number of sexual partners, history of inconsistent or no condom use, or sex work)
- Report of having sex with a man or men
- Smartphone ownership
- Self-report of being HIV negative or having an unknown status
- Self-report of having tested negative for syphilis or having an unknown status
- Not having been tested for HIV or syphilis in the previous 3 months
- Understanding the limitations of the duplex lateral flow test and the mLab+ app (eg, a confirmatory test is needed, and the self-test must be performed in the presence of a qualified clinician)

**Exclusion criteria**

- Persons with a known diagnosis of HIV or syphilis
- Persons for whom the investigators determine that participation may be detrimental to the participant or to the study (eg, those with severe cognitive deficit)
- Persons diagnosed with systemic lupus erythematosus as their medical condition could affect the results of the DPP HIV-Syphilis test
- Persons unable or unwilling to provide consent for study participation

### Ethical Considerations

All study activities were approved by the Columbia University Institutional Review Board under approval AAAU3380 prior to the recruitment or enrollment of study participants. This study was registered on ClinicalTrials.gov (NCT06059443). Study participants signed electronic informed consent forms through REDCap (Research Electronic Data Capture; Vanderbilt University) that stated the procedures for both the intervention and control arms of the study. The consent form outlined analysis procedures following the study’s completion, including the individuals and agencies who had permission to look through research records, the deidentification of all pieces of information that may identify them, and the use of their deidentified data for future research purposes. The consent form also included a statement of confidentiality. This detailed authorization for Columbia University Irving Medical Center, the New York-Presbyterian Hospital, and the principal investigator and her staff to use and disclose protected health information (PHI) in connection with the research study; an explanation of what PHI consists of; and for what purposes the PHI may be used or disclosed. Furthermore, it stated that we obtained a certificate of confidentiality from the National Institutes of Health. Participants were compensated with US $40 for their baseline appointment and US $60 for their 3-month follow-up appointment.

### Study Procedures

#### Baseline Visit

At the baseline visit, participants received HIV and STI risk reduction counseling, a box of condoms, a PrEP assessment, and referral information for clinics that provide PrEP. Participants then completed a survey via REDCap, a web-based survey software, that included questions on demographics; sexual risk behaviors, including number of sexual partners (ie, number of people they engaged in anal or oral sex with) and condomless anal intercourse within the previous 3 months; HIV and STI testing history; opinions regarding HIV and STI testing; postexposure prophylaxis and PrEP use and adherence; drug and alcohol use [27]; and HIV risk index [28]. Study staff were available to answer any questions while the participants were taking the survey.

Following completion of the survey, participants were instructed on how to log into and use the mLab+ app (Figure 1) by study staff. Next, participants verbally completed a DPP HIV-Syphilis testing assessment to ensure that they were aware of the functions and limitations of the test.

Participants completed the DPP HIV-Syphilis test guided by the mLab+ app with step-by-step images and instructions on how to complete the test. The DPP HIV-Syphilis assay, assigned a moderate level of complexity, is not yet approved by the Food and Drug Administration (FDA) for self-administration. As a result, participants completed their self-tests at the NPG clinic under the supervision of a trained clinician who was qualified to use the device according to the approved instructions for use. If the participant was unable to complete the test on their own, the clinician helped administer and interpret the test.

Testing using a DPP HIV-Syphilis kit required that the participant obtain a small drop of blood using a sterile lancet and sample loop and insert it into the SampleTainer bottle that was prefilled with a buffer solution. Participants then applied 2 drops of the sample and buffer from the SampleTainer bottle to the test. After a 5-minute wait, 4 drops of the DPP running buffer solution were added to the test by the participant. After a 10-minute waiting period, the test was ready for analysis first by the mLab+ app.

Participants took a picture of their diagnostic test on the app, which was then processed through automated image analysis software. Next, participants attached the included DPP Micro Reader, a small optical device provided with the testing kits, to the cassette to interpret the test results as described in the manufacturer’s instructions. Once the DPP Micro Reader finished running the test, participants reported the DPP Micro Reader results on the app and then were directed to a result summary screen on their mobile devices. The test took approximately 20 minutes to complete. Once complete, the result summary simultaneously displayed the results of the HIV and syphilis test.

After finishing the test, a study team member discussed the test results with the participants. Test results were securely transmitted and stored in a secure database. Participants with a positive test result were referred to care and offered confirmatory testing at the clinic.

#### 3-Month Follow-Up Visit

At the 3-month follow-up visit, participants returned to the NPG clinic for a survey and testing using the DPP HIV-Syphilis assay guided by the mLab+ app. The 3-month follow-up survey included the same measures as the baseline survey with the addition of usability assessments. The Health Information Technology Usability Evaluation Scale (Health-ITUES) and Post-Study System Usability Questionnaire (PSSUQ) were used to evaluate the mLab+ app in its ability to facilitate access to health information and guide users through testing. The Health-ITUES is a 20-item customizable instrument with 4 subscales: quality of work life (items 1‐3), perceived usefulness (items 4-12), perceived ease of use (items 13-17), and user control (items 18‐20), all scored from 1 (“strongly disagree”) to 5 (“strongly agree”) [29]. The PSSUQ is a 16-item instrument divided into 3 subscales: system usefulness (items 1-6), information quality (items 7-12), and interface quality (items 13-15), all scored from 1 (“strongly agree”) to 7 (“strongly disagree”) [30]. Both scales assess user satisfaction and perceptions of the system’s usability and have strong evidence of reliability and content and construct validity [29,30]. Participants self-administered the DPP HIV-Syphilis test at the clinic in the presence of a clinician following the same procedures as for baseline testing.

#### Study Outcomes and Measures

The primary outcome was the number of participants who were able to self-administer the DPP HIV-Syphilis test using the mLab+ app. A successful self-test was defined as having completed the DPP HIV-Syphilis test by themselves and having accurately identified and interpreted their HIV and syphilis statuses as determined by the supervising clinician. If participants were unable to self-administer the test, were apprehensive, or incorrectly used the DPP HIV-Syphilis test as per the instructions for use, the clinician intervened and helped the participants with using the test. If the clinician, rather than the participant, administered the DPP HIV-Syphilis test, the test was not recorded as a successful self-test. A research staff member from Columbia University was always present with the study participants. The primary outcome was recorded by staff running the visit, who indicated through REDCap whether the participants successfully completed the testing procedures on the app without the help of a clinician.

In addition, we measured the feasibility of this tool through several approaches: recruitment pace, retention over 3 months, usability of the app, and paradata. Paradata allowed us to quantify participants’ use of the app and are considered “free” in that they are collected in the background of app use and do not require any additional effort from the user [31]. The following paradata were collected: (1) unique participant code, (2) page accessed, (3) action performed on the page, and (4) time stamp. From these data, derivative metrics were calculated for each user and in aggregate, including duration on each page, page progression through the app, time from log-in to result, and total time from log-in to log-out.

## Results

### Sample Characteristics

**Figure 2.**
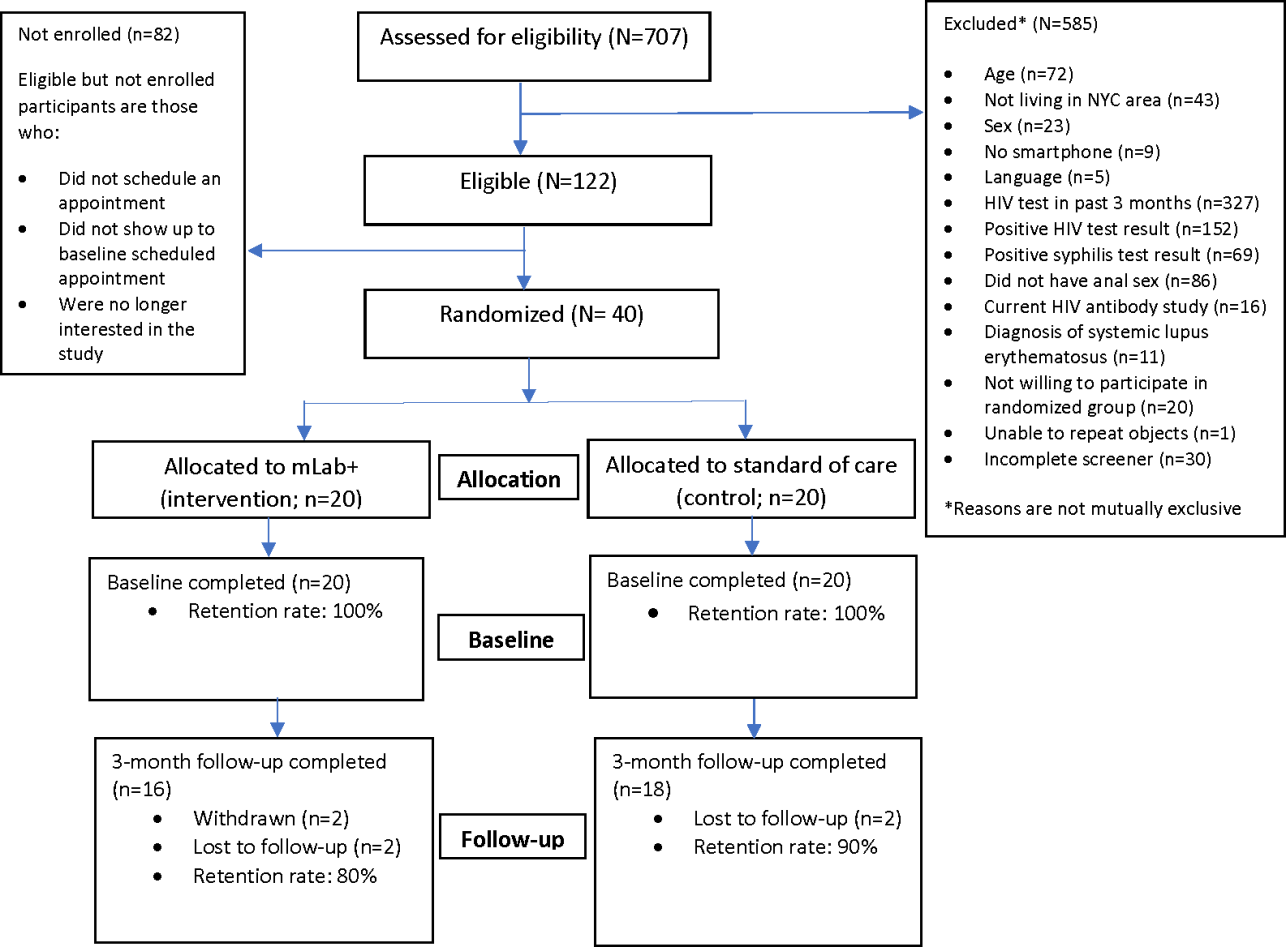
CONSORT (Consolidated Standards of Reporting Trials) diagram. This figure includes the number of total participants assessed for eligibility, randomized, and enrolled. NYC: New York City.

Of a total of 707 people who filled out an eligibility screener, 122 (17.3%) were screened as eligible, and 40 (5.7%) were enrolled and randomized to the control (n=20, 50%) or intervention (n=20, 50%) arms (Figure 2).

In total, 95% (19/20) of the participants who used the mLab+ app identified as male, and 5% (1/20) identified as nonbinary. Age ranged from 21 to 35 years, with an average of 28 (SD 3.94) years. Participants reported their race as Asian (5/20, 25%), Black (6/20, 30%), White (8/20, 40%), and other (1/20, 5%) and their ethnicity as not Hispanic or Latino (14/20, 70%), Dominican (2/20, 10%), Mexican (1/20, 5%), Puerto Rican (2/20, 10%), and another Hispanic identity (1/20, 5%).

### Primary Outcome

Most participants (16/20, 80%) were able to complete the test on their own at both time points. Table 1 shows the number of participants who completed testing independently (ie, “yes”) and with the help of a clinician (ie, “no”). Table 2 shows the test results for both HIV and syphilis at each time point.

**Table 1.** Participants who conducted the HIV and syphilis test on their own at baseline and the 3-month follow-up while using the mLab+ app at the clinic.

	Participants, n/N (%)
Baseline	
Yes	16/20 (80)
No	4/20 (20)
3-month follow-up^a^	
Yes	12/15 (80)
No	3/15 (20)

a One of the participants who was retained at the 3-month follow-up visit did not complete testing at this time point.

**Table 2.** Reactive and nonreactive HIV and syphilis test results among young men who have sex with men on the mLab+ app study at both study time points (baseline and 3-month follow-up).

	Participants, n/N (%)
Baseline	
Nonreactive for HIV and nonreactive for syphilis treponemal test	15/20 (75)
Nonreactive for HIV and reactive for syphilis treponemal test	3/20 (15)
Reactive for HIV and nonreactive for syphilis treponemal test	1/20 (5)
Reactive for HIV and reactive for syphilis treponemal test	1/20 (5)
3*-*month follow-up^a^	
Nonreactive for HIV and nonreactive for syphilis treponemal test	12/15 (80)
Nonreactive for HIV and reactive for syphilis treponemal test	3/15 (20)

a One participant did not complete testing at the follow-up visit.

#### Linkage to Care

All participants who tested reactive for HIV or syphilis at baseline (5/20, 25%) or follow-up (3/20, 15%) were referred to care. Only 40% (2/5) of those participants reported linkage to care after their baseline appointment. The others were withdrawn due to a previous HIV diagnosis (1/5, 20%), were scheduled for testing and did not show up (1/5, 20%), or did not respond to follow-up calls about linkage to care (1/5, 20%).

### Paradata

**Figure 3.**
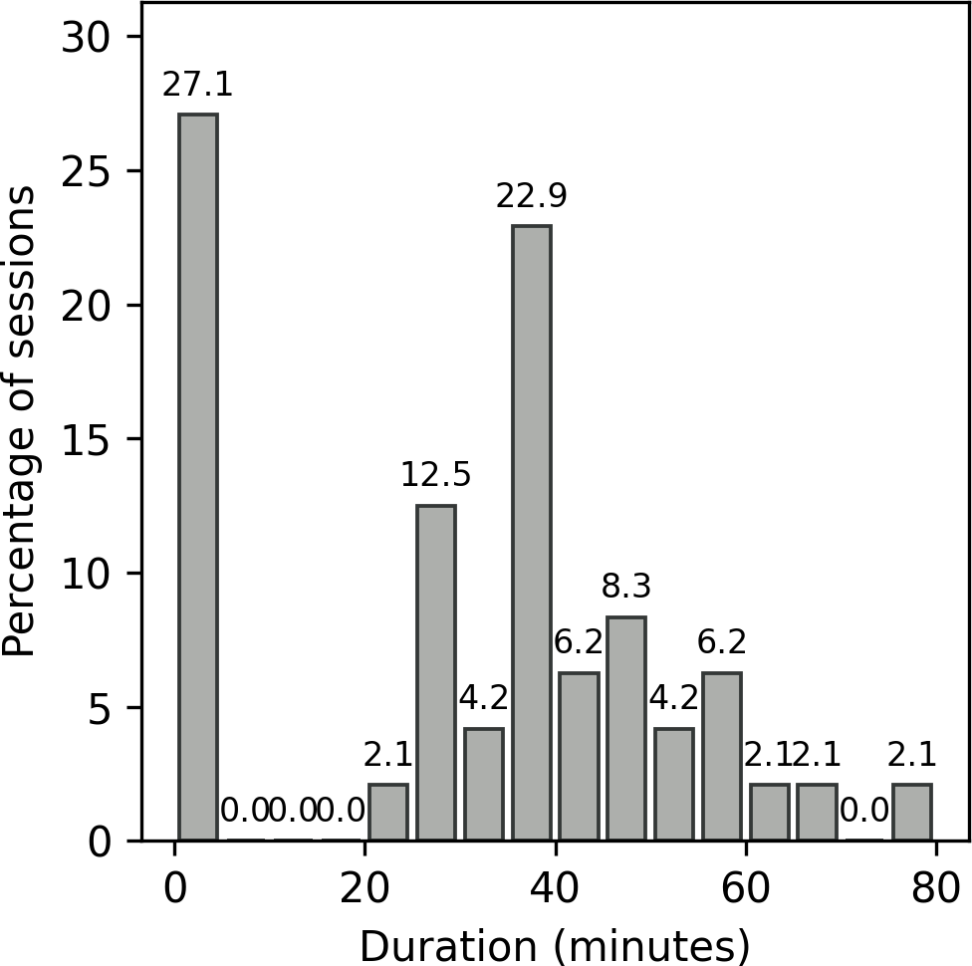
Distribution of session duration across all users enrolled in the mLab+ app study between their first visit and the 3-month follow-up visit.

**Figure 4.**
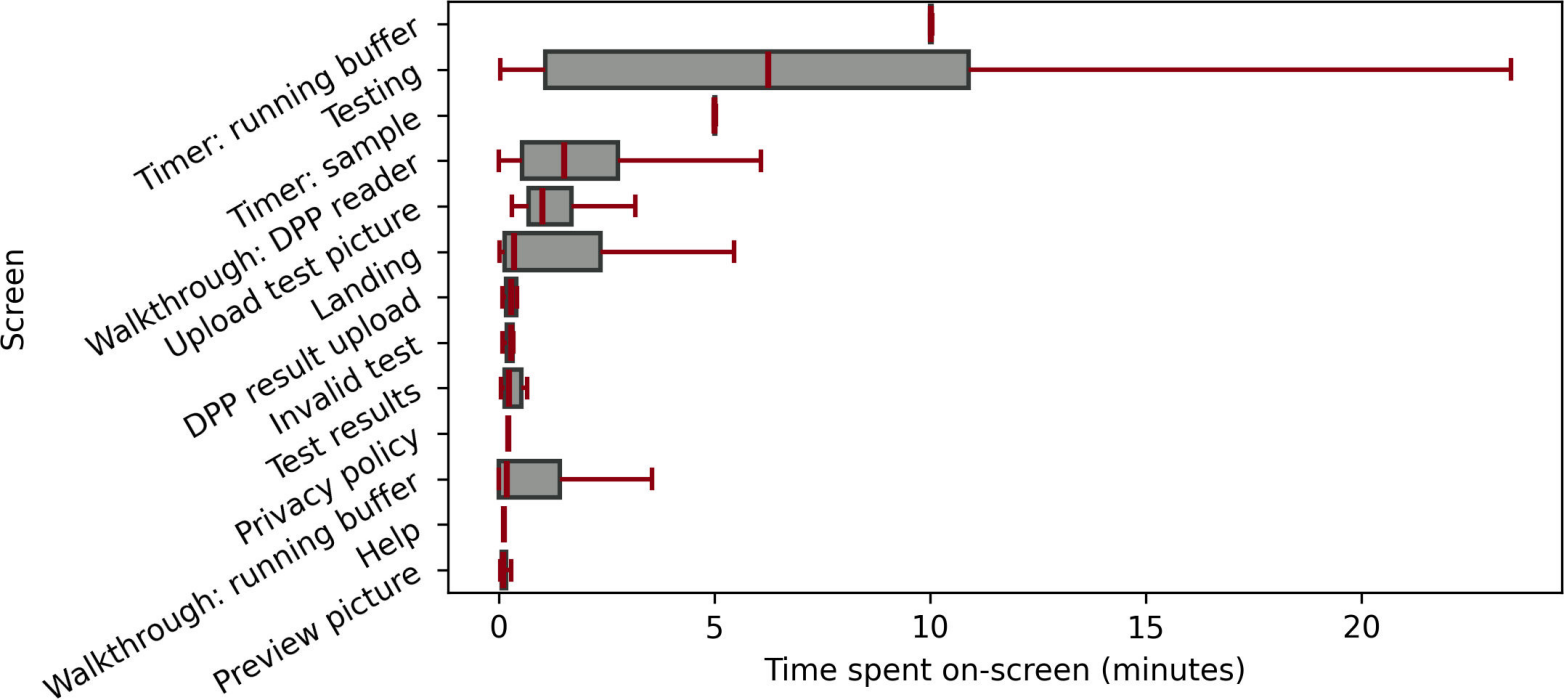
Distribution of time spent on each screen on the mLab+ app across all users during the 3-month study period (outliers greater than 1.5 times the IQR were removed for visualization purposes, but statistical calculations were not removed).

Paradata were collected during baseline and follow-up visits. The average duration of a session, from after authentication until log-out or abandonment, was 30 minutes and 33 seconds (SD 21 minutes and 40 seconds; median 36 minutes and 2 seconds; IQR 0 minutes 42 seconds-44 minutes and 15 seconds). Apart from the 27% (13/48) of sessions that were 5 minutes or less, the distribution of session durations was approximately normal (Figure 3). The distribution of duration viewing each screen (Figure 4) shows that users spent the longest time viewing testing screens (ie, timer screens, the initial testing screen, the test guided walkthroughs, test results, and picture and result upload). Auxiliary screens (ie, privacy policy and help) were typically only briefly visited. Notably, the 2 timer screens (“Timer: running buffer” and “Timer: sample”) both had very narrow distributions, indicating that time spent viewing the timer screens was brief, and the automated timers functioned as expected, moving participants along the testing workflow at the completion of each timer.

As test taking was the primary objective of app use, it is informative to look at paradata related to the testing pathway. The workflow conversion funnel (Table 3) shows the percentage of time during which a screen was visited in an individual session. These data indicate that 77% (37/48) of sessions resulted in a user visiting the screen where a test could be initiated (“Testing”) and 71% (34/48) of sessions started a test. There was no drop-off in conversion until the “Preview picture” screen, which 67% (32/48) of the sessions reached. This conversion remained constant using the DPP Micro Reader and only decreased to 63% (30/48) of sessions that ended with a test result.

**Figure 5.**
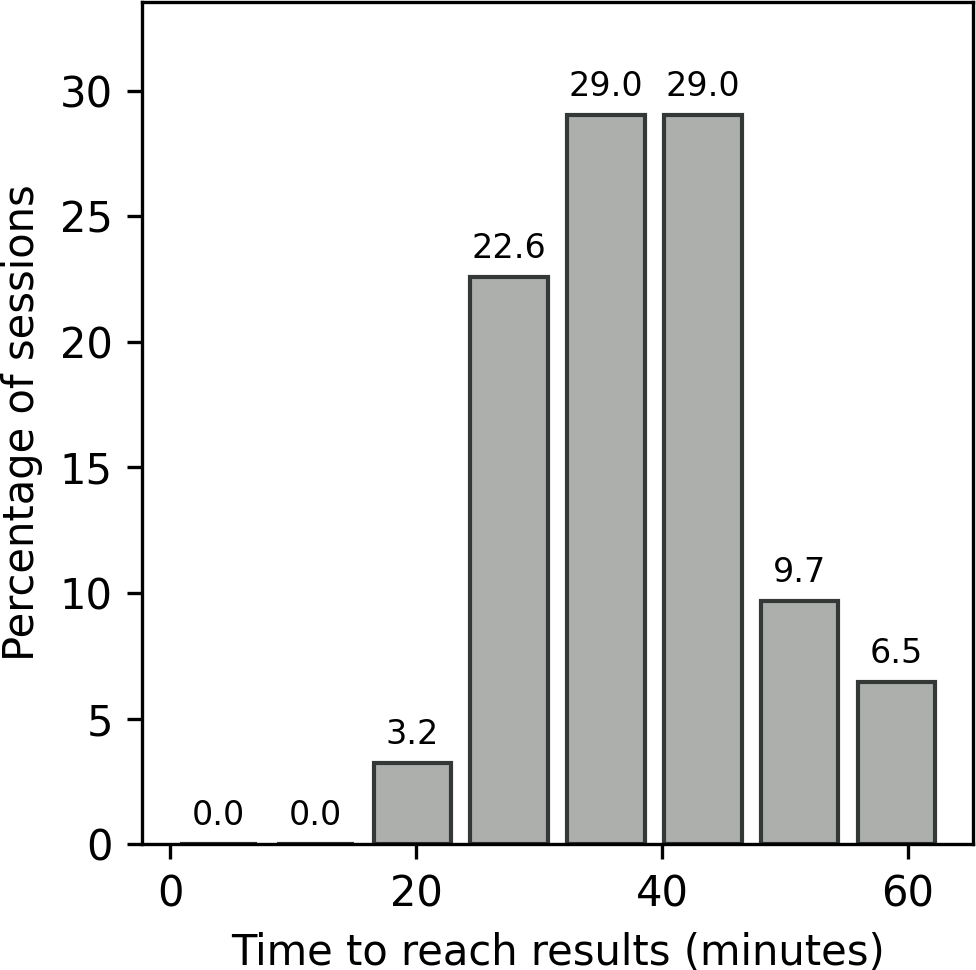
The distribution of time to reach the HIV and syphilis result screen from the first screen across all mLab+ app participant sessions that resulted in a completed HIV and syphilis test at both the baseline and 3-month follow-up visits.

Of the sessions that resulted in a completed test, the distribution of the time it took from log-in to obtaining the test results (Figure 5) was approximately normal (mean 39 minutes and 6 seconds, SD 10 minutes and 1 second; median 38 minutes and 14 seconds).

**Table 3.** Conversion rate for each mLab+ app screen, that is, the percentage of time each screen was visited in an individual session across all users enrolled in the mLab+ app study at baseline and the 3-month follow-up.

Step	Screen	Screen sessions (n=48)	Conversion rate (%)
1	Landing	47	97.92
2	Testing	37	77.08
3	Timer: sample	34	70.83
4	Walkthrough: running buffer	34	70.83
5	Timer: running buffer	34	70.83
6	Upload test picture	34	70.83
7	Preview picture	32	66.67
8	Walkthrough: DPP Micro Reader	32	66.67
9	DPP result upload	32	66.67
10	Test results	30	62.50

### Usability

The mLab+ app was rated by participants in the intervention arm using the Health-ITUES [29] and PSSUQ [32] (Table 4). The overall mean score on the Health-ITUES was 3.62 (SD 1.07; range 1-5), with higher scores indicating greater perceived usefulness. The overall mean score on the PSSUQ was 2.65 (SD 1.06; range 1-4.19), with higher scores indicating lower perceived usefulness. The scores on both measures indicate medium to high usability.

**Table 4.** Health Information Technology Usability Evaluation Scale (Health-ITUES) and Post-Study System Usability Questionnaire (PSSUQ) usability scores as rated by participants who used the mLab+ app.

Instrument	Score, mean (SD)
PSSUQ (strongly agree=1 to strongly disagree=7)	
Overall	2.65 (1.06)
System usefulness	2.51 (1.15)
Information quality	2.77 (1.03)
Interface quality	2.67 (1.24)
Health-ITUES (Strongly agree=5 to strongly disagree=1)	
Overall	3.62 (1.07)
Quality of work life	3.52 (1.08)
Perceived usefulness	3.64 (1.23)
Perceived ease of use	3.76 (1.05)
User control	3.46 (1.07)

### Recruitment

During the recruitment and enrollment period, 707 people were screened for eligibility, with 82.7% (585/707) being screened as ineligible and 17.3% (122/707) being screened as eligible. Having had an HIV test within the previous 3 months (n=327, 46.8%) and an HIV diagnosis (n=152, 21.5%) were the most frequently cited reasons for ineligibility. Exclusion criteria were not mutually exclusive. In addition, 7% (6/82) of the people who were screened as eligible did not show up to their study visit, 9% (7/82) of the participants showed up to their appointment but were found to be ineligible and not enrolled, and others (69/82, 84%) never scheduled a baseline appointment. In total, 50% (20/40) of the enrolled participants were randomized to the control arm and, thus, did not use the mLab+ app. During the study’s 6-month recruitment and enrollment period, an average of 5 participants (SD 4.57) were enrolled per month.

### Retention

Of the 20 participants who enrolled in the study and used the mLab+ app, 16 (80%) completed their 3-month follow-up study assessment. One participant had a known HIV diagnosis before enrolling in the study and was administratively withdrawn. Another participant voluntarily withdrew from the study. The remaining 2/4 (50%) were lost to follow-up.

## Discussion

### Principal Findings

The findings of this study focused on the feasibility and usability of the mLab+ app. Most, but not all, participants (16/20, 80%) were able to use the test on their own without the assistance of the clinician. This points to some of the challenges for consumers using these tests at home without the supervision of a clinician. Notably, resistance to finger stick blood collection is often a barrier to self-testing [33]. Nonetheless, past work has similarly demonstrated difficulties among people using at-home testing, such as the OraQuick HIV test, which requires fewer steps than the DPP HIV-Syphilis test and has already been FDA approved for at-home use [9]. Future studies should collect data on participant self-testing history, including experience with and acceptance of at-home tests, which may highlight additional barriers associated with successful completion of a self-test through the mLab+ app.

The overall usability of the app was medium to high, with lower scores on the usefulness subscale. Given that the mLab+ app was originally designed for in-home use, these scores reflect the use of this app in a clinical setting, which was not a part of its intended design. Furthermore, the usefulness of the app after the study visit was limited given that the app was designed to support using the HIV and syphilis test at home and participants did not have access to the test in their homes.

The recruitment and retention rate were acceptable within the context of this small sample size and would warrant further consideration in a fully powered trial. Retention would likely be improved in a future trial if research visits took place remotely [7,34], as is evidenced by the original mLab study, which largely took place over Zoom and resulted in a retention rate of 89.5% at the 6-month follow-up and 84.6% at the 12-month follow-up; this trial was much larger (N=525) and longer (12-month study period) than the mLab+ app study, demonstrating the potential for improved retention in a remote setting [7,34]. Given the FDA restrictions at the time of this study’s start, we were not able to conduct the mLab+ app study remotely. Furthermore, to improve linkage to care among HIV-positive participants, future studies should consider integrating nearby resources, including addresses and phone numbers, into the app, as well as next steps to facilitate making a physician’s appointment, as demonstrated in the SMARTtest mobile health study for HIV and syphilis testing [35]. This feature would be helpful for participants who refused on-site testing the day they received their reactive result.

Finally, the paradata highlight key aspects of user behavior and the effectiveness of the testing workflow. Users predominantly spent time on testing-related screens, such as timers, guided walkthroughs, and result screens, reflecting the primary purpose of the app. Auxiliary screens such as the privacy policy and help were visited briefly, which is expected given the scope of the study; these screens, particularly the help screen, would be expected to have more use in a larger trial or in an environment where study team members were not immediately available. The consistency in time spent on both timer screens suggests that the automated workflows function as intended, guiding users smoothly through the testing process by closely following the manufacturer’s guidelines. Importantly, the analysis of the workflow conversion funnel indicates minimal drop-off once a test was initiated. This, particularly when combined with the usability results, suggests that the app effectively supported users in completing the testing process. Furthermore, the time to complete the tests aligns well with the expected duration, although slight deviations may occur due to session resets when users resume testing after logging out. Overall, the findings underscore the usability and robustness of the app in facilitating the testing workflow and its suitability for use in a larger study.

### Limitations

This study was designed as a pilot RCT, but due to the limitations of conducting the study in a clinical setting, we are only reporting on the intervention arm, who had access to the mLab+ app and the combination test for HIV and syphilis. Although participants were randomized into intervention versus control arms, we did not include control arm data in this paper. As originally designed, study participants would use the mLab+ app and the combination test for HIV and syphilis for 3 months in a pilot RCT. The original conceptualization of the study design was to assess testing uptake and referral to services in the participants who were given access to the mLab+ app and HIV and syphilis test as compared to a control arm, which would only receive information on HIV and syphilis testing services. The FDA denied the request for study participants to use the combination test for HIV and syphilis in their homes, so the study design was modified, and participants were only able to use the test in a clinical setting under the supervision of a nurse practitioner. Future studies should consider the feasibility and acceptability of the mLab+ app outside of the clinical setting.

### Conclusions

Findings from this study support the use of the mLab+ app as a tool for assisting consumers in self-testing for HIV and syphilis. This is especially important in the context of the growing HIV and syphilis epidemic among MSM in the United States. Nonetheless, the limitations of the study design warrant further examination outside of the clinic setting to better understand the utility of these tools for improving consumer health outcomes.

## Supplementary material

10.2196/72955 Checklist 1 CONSORT-EHEALTH checklist (V 1.6.1).

## Abbreviations

CONSORT-EHEALTH: Consolidated Standards of Reporting Trials of Electronic and Mobile Health Applications and Online Telehealth
FDA: Food and Drug Administration
Health-ITUES: Health Information Technology Usability Evaluation Scale
MSM: men who have sex with men
NPG: Nurse Practitioner Group
PHI: protected health information
POC: point of care
PrEP: preexposure prophylaxis
PSSUQ: Post-Study System Usability Questionnaire
RCT: randomized controlled trial
REDCap: Research Electronic Data Capture
STI: sexually transmitted infection
